# Molecular pathology of thymomas: implications for diagnosis and therapy

**DOI:** 10.1007/s00428-021-03068-8

**Published:** 2021-03-05

**Authors:** Alexander Marx, Djeda Belharazem, De-Hyung Lee, Zoran V. Popovic, Christoph Reißfelder, Berthold Schalke, Sebastian Schölch, Philipp Ströbel, Cleo-Aron Weis, Yosuke Yamada

**Affiliations:** 1 Institute of Pathology, University Medical Centre Mannheim and Medical Faculty Mannheim, Heidelberg University, Theodor-Kutzer-Ufer 1-3, 68167 Mannheim, Germany; 2 Department of Neurology, University of Regensburg, Regensburg, Germany; 3 Department of Surgery, University Medical Centre Mannheim and Medical Faculty Mannheim, Heidelberg University, Mannheim, Germany; 4 Junior Clinical Cooperation Unit Translational Surgical Oncology (A430), German Cancer Research Center (DKFZ), Heidelberg, Germany; 5 Institute of Pathology, University Medical Center Göttingen, University of Göttingen, Göttingen, Germany; 6 Department of Diagnostic Pathology, Kyoto University Hospital, Kyoto, Japan

**Keywords:** AIRE, Microsatellite instability, MicroRNA, SMARCA4, Myasthenia gravis, Immune checkpoint inhibitors

## Abstract

Thymomas exhibit a unique genomic landscape, comprising the lowest on average total mutational burden among adult human cancers; a unique point mutation in the *GTF2I* gene in WHO type A and AB thymomas (and rarely others); almost unique *KMT2A-MAML2* translocations in rare WHO type B2 and B3 thymomas; a unique *YAP1-MAML2* translocation in almost all metaplastic thymomas; and unique miRNA profiles in relation to *GTF2I* mutational status and WHO histotypes. While most thymomas can be diagnosed solely on the basis of morphological features, mutational analyses can solve challenging differential diagnostic problems. No molecular biomarkers have been identified that predict the response of unresectable thymomas to chemotherapy or agents with known molecular targets. Despite the common and strong expression of PDL1 in thymomas, immune checkpoint inhibitors are rarely applicable due to the poor predictability of common, life-threatening autoimmune side effects that are related to the unrivaled propensity of thymomas towards autoimmunity.

## Introduction

Thymomas constitute the largest subgroup (75–80%) among thymic epithelial tumors (TETs) and are the focus of this review. Thymic carcinomas (TCs) and thymic neuroendocrine tumors that constitute 10–20% and 1–2% of TETs, respectively, are reviewed elsewhere (Ströbel et al., this volume and in [1, 2]). Taking the content of immature thymocytes and the morphology of tumor cells into account, TCs are distinguished from thymomas which, in turn, are separated into WHO type A, AB, B1, B2, B3,and metaplastic thymomas as well as “micronodular thymoma with lymphoid stroma” (MNT) [3]. Apart from MNTs, TETs are malignant cancers with variable metastatic potential that increases from type A and AB, through B1, B2, and B3 thymomas to the most aggressive TCs [3]. Staging of TETs should follow the recently developed TNM system that is gradually replacing the Masaoka-Koga system [4]. Since key therapeutic guidelines still refer to the Masoaka-Koga system [5], both staging systems are still commonly used in parallel [4]. In terms of therapy, the prime aim is complete tumor resection that is usually the definite and only required intervention, while non-resectability often implies incurability [5]. However, adjuvant radiotherapy in case of uncertain resection, high tumor stage, or high-grade histology can rescue a significant number of patients [6]. In case of unresectable and recurrent thymomas, platinum-based chemotherapy is the empirical standard first-line treatment [5]. This review focuses on recent findings in the pathogenesis of thymomas and highlights gaps of knowledge that prevent efficient targeted treatment to date.

## Biological features of thymomas with diagnostic and therapeutic relevance

### Autoimmunity and expression of immune checkpoint molecules

Thymomas are unique tumors due to their almost consistent non-tolerogenic, intratumorous thymopoiesis that is almost never encountered in TCs and not in other carcinomas. This feature is likely the major pathomechanism that leads to the unprecedented frequency of autoimmune phenomena (about 80%) and autoimmune diseases (about 40%) in patients with thymoma but not in patients with other malignancies [7]. Among the autoimmune targets, striated muscle proteins prevail as reflected by the fact that myasthenia gravis (MG) due to autoantibodies to the Acetylcholine Receptor (AChR) and striational autoantigens (e.g., Titin, skeletal and cardiac Ryanodine Receptors (RYRs)) is the leading thymoma-associated autoimmune disease [8]. However, almost any other organ-specific (e.g., thyroid, hepatic, renal) and systemic autoimmune disease (e.g., SLE, RA) can occur either in isolation or combined with MG or other autoimmune diseases [7, 9]. The pathogenesis of most thymoma-associated autoimmune diseases is unknown. By contrast, multiomics molecular analysis revealed that thymoma-associated MG is linked to aneuploidy and over-expression of genes that encode either bona fide (e.g., AChR) or closely related (e.g., neuronal RYRs) autoimmune targets [10], while other defective tolerogenic features (e.g., the lack of *AIRE* expression [11–14] and defective intratumorous generation ofregulatory T cells [12, 15, 16]) might be permissive but not causative [10], although this is controversial [17]. Different molecular pathways may elicit MG in different thymoma histotypes [18].

The inclination of thymomas to autoimmune diseases has a bright diagnostic and dark therapeutic side: While preoperative detection of autoimmune features is a strong hint that a mediastinal mass is a thymoma, autoimmunity is a drawback in the era of immune interventions. Since thymomas are the cancers with the highest prevalence of abundant and strongly PDL1-expressing tumor cells [19], thymoma patients appear as ideal candidates for immunotherapies. Unfortunately, immune checkpoint inhibitors (ICIs) elicit severe autoimmune phenomena in most thymoma patients even if such phenomena are missing before therapy [2, 20]. Echoing the focus of thymoma-associated autoimmunity on striated muscle, the most life-threatening side effects of ICIs in thymoma patients are myositis, myocarditis, and MG [21–23]. In patients with TCs that are not “naturally” prone to autoimmunity, such side effects are less common [2, 24, 25].

### Immunodeficiency in thymoma patients

Thymoma-associate acquired T cell and B cell immunodeficiencies are common and often a facet of autoimmunity. Good syndrome (in 5% of patients) is characterized by a near lack of B cells and hypogammaglobulinemia, variable CD4 T cell cytopenia, and impaired T cell activation [26]. Hypogammaglobulinemia results from autoreactive CD8+ T cells attacking B cell precursors in the bone marrow [27]. The mechanisms that elicit severe combined deficiency of CD4 and CD8 T cells [28] or the acquired hypoexpression of *CD247* (encoding the CD3 zeta-chain) are unclear. CD247 hypoexpression entails susceptibility to infections [26] and, hypothetically, the increased prevalence of non-thymic cancers in thymoma patients [29]. Chronic mucocutaneous candidiasis in thymoma patients results from defective Autoimmune Regulator Gene (*AIRE*) expression in thymomas [11–13]. This elicits neutralizing autoantibodies to IL17-associated cytokines and impairs cytokine-dependent macrophage activation that, in turn, is needed to keep Candia in check [30]. Thymoma-associated immunodeficiency is a diagnostic challenge and can contribute to severe morbidity and even mortality [31–34].

## The genomic landscape of thymomas

### Genetic alterations in treatment naïve common thymoma types

The Cancer Genome Atlas (TCGA) consortium reported on the genetic, transcriptomic, epigenetic, miRNA and proteomic landscape of 107 thymomas (types A, AB, B1–3, MNTs) and 10 TCs from patients without prior therapy, including a high proportion of low-stage cancers [10].

In terms of somatic copy number variations, the TCGA findings were in good agreement with historic CGH studies that revealed an overall low prevalence of genomic alterations in thymomas, with particularly rare abnormalities in type A and AB compared to B2 and B3 thymomas and TCs [35–37]. Also, gains and losses were commonly large-scale alterations such as whole chromosome or chromosome arm losses and gains, with losses of chromosome 6 material (harboring the *FOXC1* tumor suppressor gene at 6p25.3 [38]) and gains in 1q as the most common structural abnormalities across all histotypes [10, 37].

One of the most prevalent somatic mutations of thymomas is a single nucleotide hot-spot mutation (c.74146970T>A; p.L424H) in the general transcription factor IIi gene (*GTF2I*) [10, 39, 40]. It occurs in about 80% of type A and AB thymomas, while it is exceptionally found in type B thymomas and rare TC [10, 40]. Less common recurrent alterations concern gain-of-function mutations of *HRAS* (mainly in type A and AB thymomas) and *NRAS* (in type A and B thymomas), and loss-of-function mutations of *TP53* (in type B thymomas and TCs). The enrichment of C>T mutations within CpG di-nucleotides is an age-related signature [41] that fits well with the age of thymoma patients [42]. KIT mutations and oncogenic driver mutations or translocations that are characteristic of lung and other cancers have not been observed.

### Lowest total mutational burden of thymomas among adult cancers and rare MSI

**Fig. 1:**
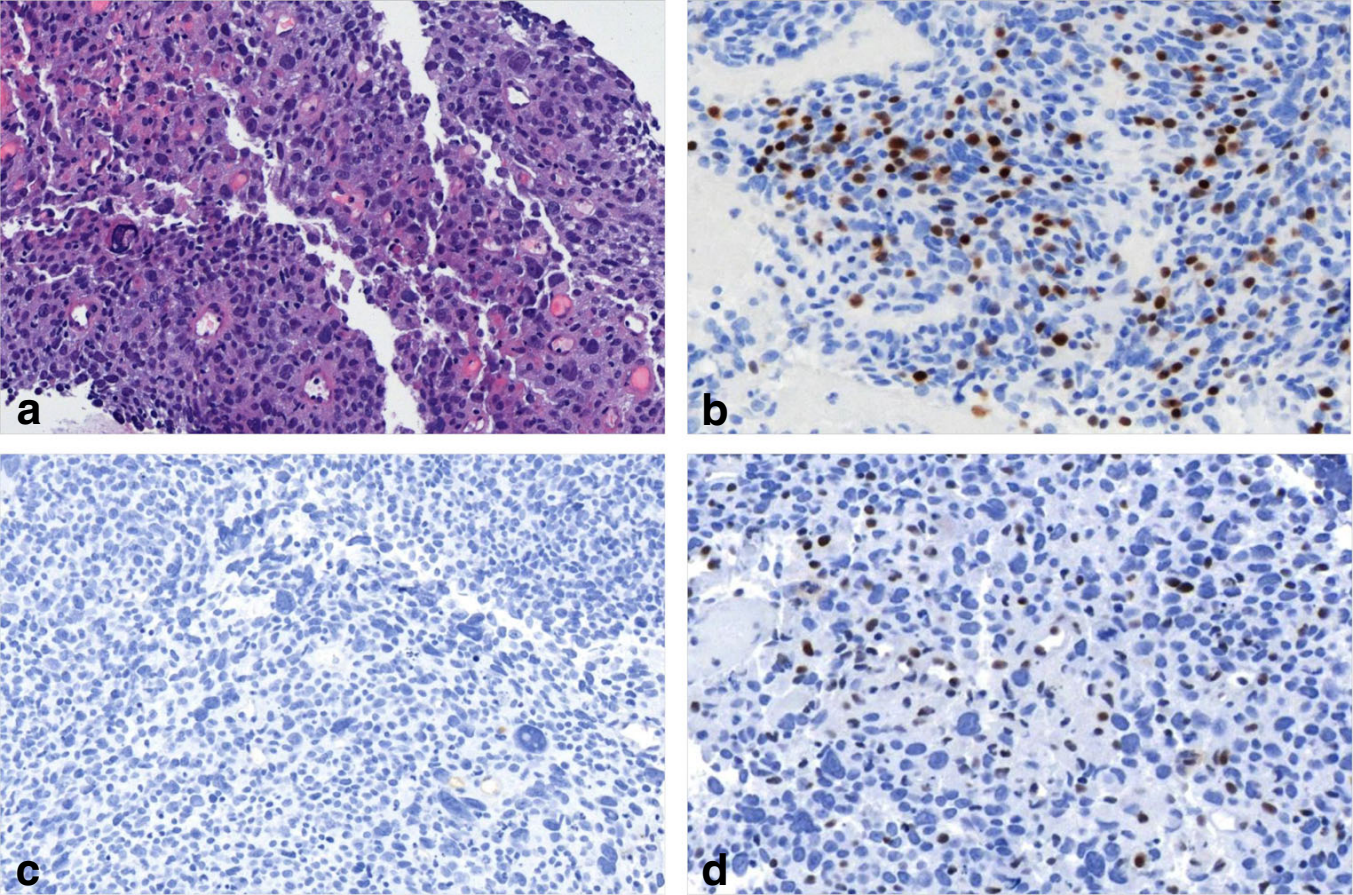
Microsatellite instability in thymoma. **a** Type B3 thymoma with anaplasia; **b** Presence of TdT expressing immature T cells; **c** Absence of CD117 expression; **d** Defective expression of MLH1 in tumor cells but not in accompanying lymphocytes. HE stain in (**a**); immunoperoxidase in **b**–**d**. (×200)

On average, thymomas exhibit the lowest total mutational burden (TMB) among all adult human cancers tested in the TCGA network [41]. While a single TC among the 10 tested carcinomas showed microsatellite instability (MSI) due to a pathogenic nonsense mutation (E37*) in the *MHL1* gene [41], none of the 107 tested thymomas exhibited this oncogenic feature. The latter observation may not be representative in light of our reference pathology experience (Fig. 1) and historic studies that revealed MSI in about 10% of thymomas using a PCR-based assay [19, 43].

### Recurrent translocations in metaplastic thymomas

**Fig. 2:**
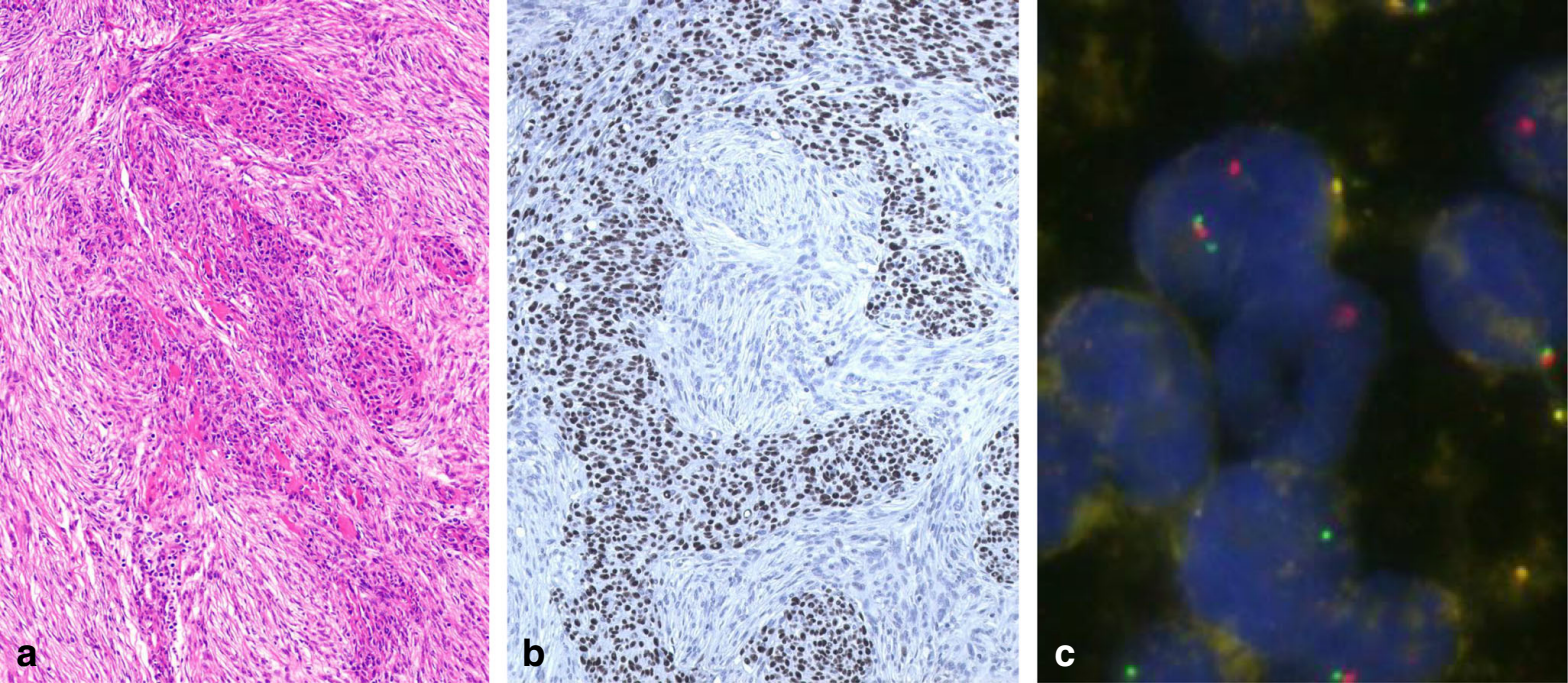
Metaplastic thymoma with recently described YAP1-MAML2 translocation. **a** Biphasic, epithelioid and spindle cell tumor; **b** Characteristic expression of p40 in the epithelioid but not the metaplastic/spindle cell component; **c** FISH analysis showing the split of the MAML2 break-apart probe (**a** HE stain, × 200; **b** immunoperoxidase, ×200; **c** immunofluorescence, ×400)

While the TCGA and other previous sequencing efforts [40, 41, 44] failed to identify recurrent translocations in type A, AB, B1–B3 thymomas and MNTs, a YAP1-MAML2 translocation (with two distinct fusion products) was recently detected by DNA RNA sequencing in all six metaplastic thymomas successfully tested so far [45]. These cases were chemotherapy naïve in accordance with their generally indolent clinical behavior [45]. Although the spindle cell component of this biphasic thymoma type (Fig. 2) vaguely resembles spindle cell areas in type A and AB thymomas [3], GTF2I mutations were consistently absent [45]. The functional effects of the YAP1-MAML2 fusion gene have not been studied but are likely oncogenic.

### Recurrent translocations in rare type B2 and B3 thymomas

Recurrent *KMT2A-MAML2* translocations were recently identified in 6% of clinically aggressive type B2 and B3 thymomas and a single case of combined TC (B3 thymoma with small TC component) [46]. The translocations variably involved exons 8, 9, 10, or 11 of *KMT2A* and exon 2 of *MAML2*, and are highly characteristic of type B2 and B3 thymomas, because they were previously found only in very rare leukemias, myelodysplastic syndromes, and one plasmacytoma but not in any other tumor among over 250.000 cases sequenced by Foundation Medicine, including 266 thymic carcinomas [46]. The function of the respective fusion proteins in thymomas is currently unclear, but might be oncogenic drivers, since 7 of the 11 cases did not harbor any concurrent mutations, while the four others showed only single additional mutations/variants in *TP53*, *ARID1A, SFB1*, and the *TERT* promoter [46]. Furthermore, KMT2A (also known as MLL) is a known oncogenic driver in translocations with other partner genes in sarcomas [47] and leukemias [46]. Since the index case of the series of Massoth et al. was a recurrent B3 thymoma biopsied after chemotherapy, and the treatment status of the other cases was not reported, it is currently unclear, whether the *KMT2A-MAML2* translocation is an early or late molecular event. The latter possibility would explain why the fusion was missed in the TCGA series.

### SMARCA4-deficient mediastinal/pulmonary tumors

**Fig. 3:**
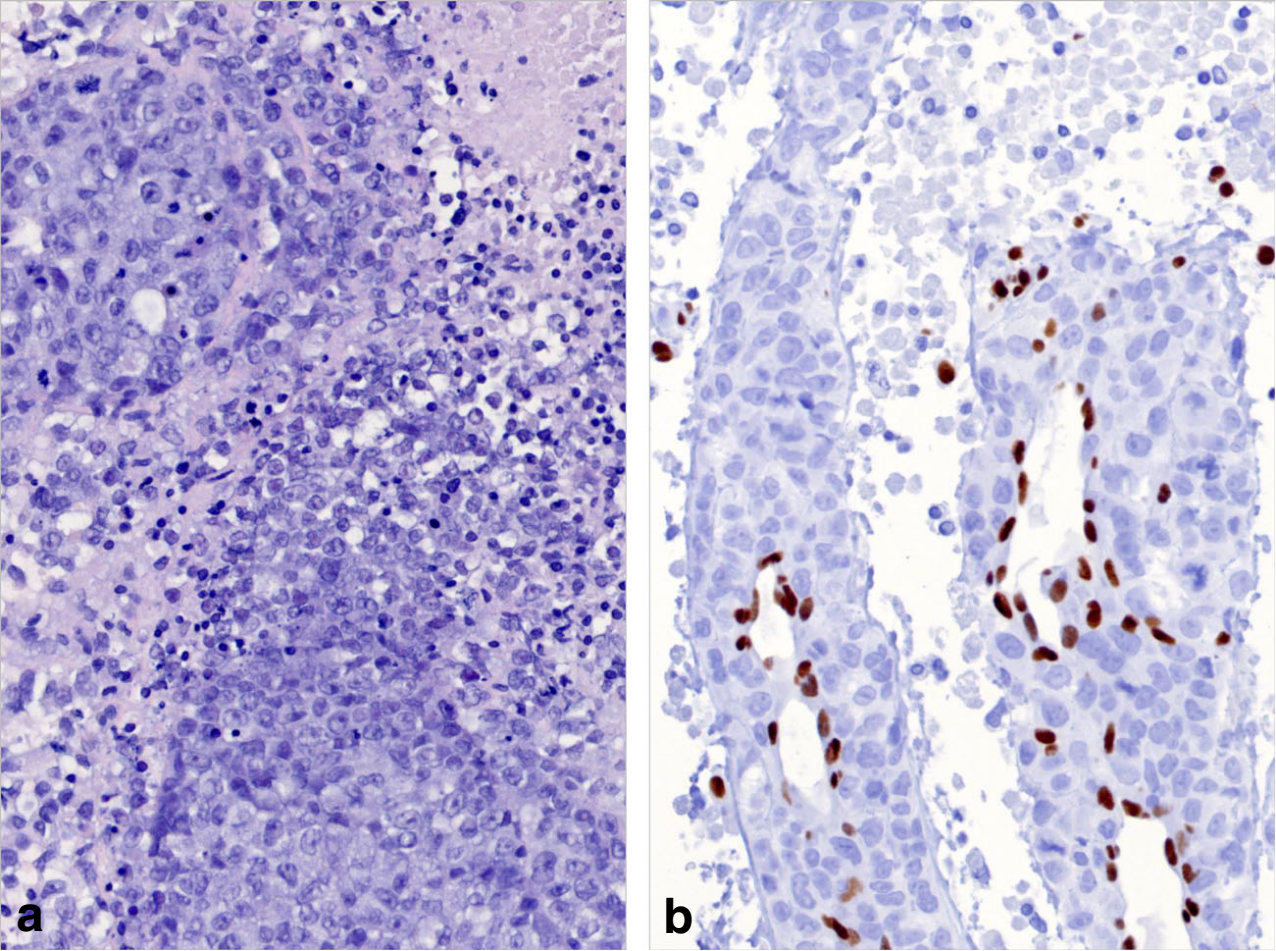
SMARCA4-deficient thoracic tumor; core needle biopsy of a mediastinal mass involving the lung (or vice versa). **a** Partially necrotic, undifferentiated tumor composed of large, poorly cohesive round and polygonal cells with large nuclei and prominent nucleoli; **b** Absence of SMARCA4 expression in the tumor cells, strong expression of SMARCA4 in endothelial cells. An identical staining pattern was seen with an antibody to SMARCA2 (not shown) (**a** HE stain, ×350; **b** immunoperoxidase)

SMARCA4-deficient cancers are a new cancer type in the upcoming WHO classification of thoracic tumors. They commonly show pleomorphic, large, and anaplastic cells, eventual deficiency of keratins, common necrosis, and defective expression of SMARCA4 (Fig. 3) or combined SMARCA4/SMARCA2 deficiency [48, 49]. So far, no bona fide thymus-restricted case has been reported, while co-invasion of thymus and lung is not uncommon. SMARCA4-deficient cancers may be confused with “thymomas with anaplasia” (see Fig. 1) that can also show defective keratin expression [50] but retain SMARCA4 expression (own observation). Since SMARCA4-deficient tumors commonly express SOX2 and SALL4 [51], mediastinal germ cell tumors also enter the differential diagnosis.

### Micro-RNAs in thymomas

Micro-RNAs (miRNAs) are non-protein-coding RNAs regulating post-transcriptional gene expression in many cancers [52], thymus development [53] and thymoma-associated autoimmunity [53–55]. Transcriptional overexpression of a large miRNA cluster on chromosome 19q13.42 (termed C19MC) is a common feature of type A and AB thymomas [10, 41] and associated with activation of the PI3K/AKT/mTOR pathway. Therefore, respective inhibitors might be therapeutic options in rare cases of unresectable type A and AB thymomas [41]. Another large cluster on chromosome 14 (C14MC), supposedly with tumor suppressor function, is transcriptionally silenced in many TCs [56]. In addition, various non-clustered single miRNAs are differentially expressed between thymomas and TCs [56, 57] and thought to contribute to the tumorigenesis of TCs (reviewed in [2]). So far, miRNAs do not play a role as diagnostic or therapeutic targets, and, unlike in renal cancers [58], have not been evaluated as predictive biomarkers (e.g., for sunitinib resistance).

### The integrated landscape of treatment-naïve thymic epithelial tumors

**Fig. 4:**
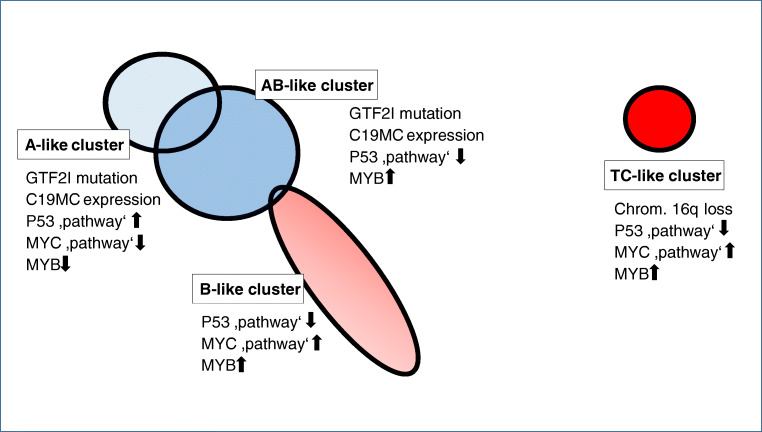
Integrated genomic landscape of thymomas and thymic carcinomas according to The Cancer Genome Atlas analysis (modified from Radovich et al. [10]). Cohorts comprise samples that are placed in the map according to similarities in their genomic profiles using all molecular platforms. The substantial overlap between the A-like and AB-like cohort indicates that quite some WHO type A and AB thymomas occur in either cohort, suggesting a molecular continuum. Little overlap between the B-like and the AB-like cohorts; of ten thymic carcinomas, one case with unique molecular features (including lack of the typical loss of 16q) was “misplaced” in the AB-like cluster. A selection of key differentially expressed molecular features is listed with each cluster. C19MC denotes a large micro-RNA cluster on chromosome 19q13.42

Integrating TCGA data from the analysis of somatic copy number alterations, mRNA, miRNA, DNA methylation, and reverse phase protein arrays of all TETs using a “cluster-of-cluster” algorithm separated the thymomas into 3 molecular subtypes that were distinctly different from the tightly clustering TCs. As shown in Fig. 4, there was a significant overlap between the A-like and the AB-like cluster, while the members of the B-like cluster formed a continuum with minimal overlap with the AB-like cluster [10]. In agreement with previous findings [40], the GTF2I mutation was largely restricted to the A-like and AB-like clusters. In addition, the clusters segregate with the expression of key oncogenes (e.g., *MYC/MAX* and *MYB*) and suppressor genes (*TP53*), lymphocyte content, WHO histotype, prognosis, and MG status [10], providing strong evidence for the relevance of the WHO histological classification.

### Genetic alterations in thymomas following chemotherapy

The TETs investigated by the TCGA consortium were chemotherapy-naïve to avoid poorly interpretably secondary genomic alteration in view of poorly standardized adjuvant therapies used to date [41]. On the other hand, targeted therapies in TETs are typically considered after the failure of various first-line treatments, making the study of post-chemotherapeutic TETs by Wang et al. even more compelling [44]. However, no recurrent genetic alterations were identified even in heavily pretreated thymomas, while TC frequently showed mutations in potential oncogenic driver genes with a role in chromatin remodeling (e.g., *SMARCA4*), histone modification (*BAP1, SETD2, ASXL1*), and DNA methylation (*TET2, DNMT3a34, WT1*). Mutations in these genes appear worth testing as biomarkers in TCs, as they constitute promising biomarkers in advanced renal cell carcinomas treated with sunitinib, sorafenib, and everolimus [59, 60], i.e., drugs that are used in advanced TCs (reviewed in [1]). No such perspective is currently obvious in advanced thymomas that are poor responders to sunitinib [61].

## Diagnostic implications of molecular alterations in thymomas

Since most differential diagnostic problems in thymomas can be solved by morphology, the role of diagnostic molecular pathology in TETs is limited (Table 1). Exceptions may arise in small biopsies.

**Table 1:** Recurrent molecular alterations with potential differential diagnostic relevance in TETs. Radovich et al. 2018 [10]; Feng et al. 2017 [39]; Petrini et al. 2015 [12]; Viviero et al. 2020 [45]; Massoth et al. 2020 [46]

Genetic alteration	Type A thymoma	Type AB thymoma	Type B1 thymoma	Type B2 thymoma	Type B3 thymoma	MNT*	Metaplastic thymoma	Thymic carcinoma
GTF2A, p.L424H	82–100*%	71*–79%	0*–32%	0*–22%	10*–21%	50% (1 of 2*)	n.t.	0*–8%
YAP1-MAML2 translocation	(---)	(---)	(---)	(---)	(---)	(---)	100%	n.k.
KMT2A-MAML2 translocation	(---)	(---)	(---)	<10%	<10%	n.k.	n.k.	(---)
16q loss	(---)	(---)	(---)	(---)	(---)	(---)	n.t.	80%*

*Results obtained by the TCGA THYM consortium [10]

*TETs*, thymic epithelial tumors; *(---)*, 0%; *n.k.*, not known; *n.t.*, not tested

A *GTF2I* (p.L424H) mutation strongly argues for the diagnosis of type A over a focally spindly type B3 thymoma or a metaplastic thymoma [41, 45]. The distinction of atypical type A thymomas and polygonal cell-rich type A thymomas “with neuroendocrine morphology” [62] from type B3 thymomas may be other rare indications for molecular testing.

*YAP1-MAML2* translocation testing is usually not necessary to diagnose metaplastic thymoma, if the biphasic nature, p40(−) spindle cell component and lack of immature T cells are taken into account [45]. Whether the derivation of some sarcomatoid carcinomas from metaplastic thymomas can be confirmed by *YAP1-MAML2* testing is unknown. In small biopsies, absence of the mutation may help to confirm rare type A and AB thymomas with extensive “fibrous bands” showing an EMA(+), actin(+), and p40(−) phenotype (own observation and [63]).

The diagnostic relevance of the recently described *KMT2A-MAML2* translocations in aggressive type B2 and B3 thymomas [46] needs confirmation.

## Therapeutic implications of molecular alterations in thymomas and perspectives

The results of the TCGA study of thymic epithelial tumors (Table 1) confirmed previous studies that revealed absence of targetable mutations as tissue-based biomarkers in thymomas (reviewed in [1, 2]) and presence of only rare clinically meaningful mutations (e.g., of the KIT gene) in TCs [64]. In line with this, “targeted” approaches that took transcriptomic or immunohistochemical findings (e.g., overexpression of supposedly unmutated genes coding for tyrosine kinases or angiogenic factors) into account achieved rather limited success (Table 2). Accordingly, interference with other oncogenic principles (like nuclear export inhibition) is currently being investigated (reviewed in [1]). Overcoming the unacceptable frequency and severity of immune checkpoint inhibitor (ICI)-induced autoimmune side effects and simultaneously maintaining ICI therapeutic efficiency is another perspective [20]. The recently described translocations, YAP1-MAML2 and KMT2A-MAML2 in rare metaplastic and type B2 and B3 thymomas, respectively, are currently not specifically targetable either [45, 46]. However, it will be important to investigate, whether the respective fusion proteins depend in a similar way on unmutated EGFR signaling for their tumorigenic function as does the CRTC1-MAML2 fusion protein in EGFR inhibitor-sensitive mucoepidermoid carcinomas [72].

**Table 2:** Targeted therapies in TET patients, including thymomas and thymic carcinomas (TCs) [61, 65–71]

Drug	Target	Response rate	TTP/PFS	Reference
Gefitinib	EGFR	4%	4 months	Kurup et al. 2005
Imatinib	KIT	0%	3 months	Palmieri et al. 2012
Belinostat	HDAC	0% and 8%*	5.8 months	Giaccone et al 2011
Saracatinib	SRC	0%	5.3 months	Gubens et al. 2015
Buparlisip	PI3K	7%	11.1 months	Zaid et al. 2018
Everolimus	mTOR	12%	10.1 months	Zucali et al. 2018
Sunitinib	KIT, VEGFR, PDGFR	6% and 26%**	7.2 months	Thomas et al. 2015
Milciclib	CDK4/6	3.3% and 4.2%***	5.6 and 5.7 months	Besse et al. 2018***

*8% in thymomas, 0% in TCs; **6% in thymomas, 26% in TC patients; ***two trials with 102 cases overall, including 37 thymomas (see also [1])

## Conclusion

Uncovering many facets of the molecular landscape of thymomas has improved our understanding of pathways with relevance for oncogenesis and autoimmunity but did not reveal targets that are vulnerable to currently available therapeutic agents. It is hoped that whole genome and ex vivo single cell sequencing, the analysis of non-protein-coding RNAs, and the development of relevant model system for high throughput drug screening will overcome the current, unsatisfactory situation and advance thymoma management into the realm of truly targeted therapies.

## Declarations

Morphological evaluation of archival material from the Institute of Pathology, University Medical Centre Mannheim, does not require patient consent as approved by the Medical Ethics Committee II, Medical Faculty Mannheim, Heidelberg University (ethics approval # 2017-806R-MA).
